# Five cases of tremorgenic mycotoxicosis in dogs – clinical aspects and analytical methods

**DOI:** 10.1007/s11259-026-11458-z

**Published:** 2026-08-11

**Authors:** Leon Hart, Christina Rehagel, Michael Kuhn, André Taube, Lukas Jozefowitz, Esther Hassdenteufel, Benedikt Cramer, Ömer Akineden, Ewald Usleber

**Affiliations:** 1 Chair of Dairy Sciences, Institute of Veterinary Food Science, Justus-Liebig-University, Ludwigstrasse 21, Giessen, 35390 Germany; 2 Faculty of Food and Nutrition Sciences, Hochschule Niederrhein University of Applied Sciences, Mönchengladbach, Germany; 3 Institute of Food Chemistry, University of Münster, Münster, Germany; 4 Clinic for Small Animals, Department of Veterinary Clinical Sciences, Justus-Liebig- University, Giessen, Germany

**Keywords:** Mycotoxins - penitrem A, Paxilline, Cyclopiazonic acid, *Penicillium crustosum*, Dog mycotoxicosis

## Abstract

Tremorgenic mycotoxicosis is a well-recognized cause of acute neurotoxicosis in dogs, most commonly associated with penitrem A (PTA), which is mainly produced by *Penicillium crustosum*. However, analytical confirmation of such cases is rare. This study describes five clinical cases of canine tremorgenic mycotoxicosis in Germany, together with comprehensive analytical confirmation. All dogs had been presented at the veterinary clinic of Justus-Liebig-University because of acute-onset generalized tremors. Clinical findings, therapeutic measures and final outcome were documented. Samples of gastric content (induced emesis or gastric lavage) and blood serum (if available) were analyzed for several neurotoxic mycotoxins, penitrems A–F, paxilline (PAX), α-cyclopiazonic acid (CPA), and ergoline alkaloids. Analysis was done by enzyme immunoassay (EIA), and by HPLC-MS/MS. EIA of gastric content was positive for PTA in all samples, and for PAX, CPA, and ergoline alkaloids in some. HPLC-MS/MS analysis confirmed the presence of penitrems A–F, PAX, and CPA. Trace levels of PTA, PAX and CPA were also detected in serum samples. Penitrem producing *P. crustosum* was isolated from gastric content of all animals. This is the first full analytical study on acute canine tremorgenic mycotoxicoses in Germany, and presents comprehensive results along the causal chain of tremorgenic mycotoxicosis. In addition to the penitrems, several other neurotoxic mycotoxins (PAX, CPA, ergoline alkaloids) may play a role in canine mycotoxicosis. While immediate stabilisation and symptomatic treatment are paramount, prompt mycotoxin testing can help identify the source of poisoning during differential diagnosis.

**Supplementary Information:**

The online version contains supplementary material available at https://doi.org/10.1007/s11259-026-11458-z.

## Introduction

Many fungal species belonging to several genera (*Penicillium* spp., *Aspergillus* spp., *Neotyphodium* spp., and others) are capable to produce a variety of tremorgenic mycotoxins (for example, aflatrem, lolitrem B, penitrems, paxilline. roquefortin C), the majority of these are indole derivatives (Frisvad et al. 2006; Reddy et al. 2019). Fungi of the genus *Penicillium* are ubiquitously distributed worldwide and are among the most common contaminants of food and animal feed (Hayes et al. 1976; Richard et al. 1981; Hocking et al. 1988; Naudé et al. 2002; Young et al. 2003; Rundberget et al. 2004; Eriksen et al. 2010, 2013). Tremorgenic mycotoxicosis both in animals and in humans is a severe neurological condition. The most potent and clinically significant tremorgenic mycotoxin is penitrem A (PTA), a lipophilic indole-diterpene that readily crosses the blood-brain barrier. Its primary producer is *Penicillium crustosum*, which frequently contaminates spoiled foods and organic waste (Eriksen et al. 2013; Lewis et al. 2005; Botha et al. 2019; Reddy et al. 2019; Wilson et al. 1968, 1972).

Dogs are particularly predisposed to mycotoxicosis due to their opportunistic and indiscriminate feeding behavior, which frequently leads to ingestion of moldy or decomposing food items. Clinical reports have repeatedly described intoxications following ingestion of contaminated substrates, such as moldy apples (Eriksen et al. 2010), walnuts (Richard et al. 1981; Munday et al. 2011; Fritz et al. 2020), cooked rice (Naudé et al. 2002), cream cheese (Richard and Arp 1979; Young et al. 2003), hamburger buns (Hocking et al. 1988), commercial dog feed, compost piles (Boysen et al. 2002; Novotna et al. 2023), and household garbage (Walter 2002). In some reports, the diagnosis of mycotoxicosis was solely presumed on the basis of clinical signs without toxicological confirmation by mycotoxin analysis (Fritz et al. 2020; Braun et al. 2024).

Next to acute onset of generalized tremor, clinical symptoms of tremorgenic mycotoxicosis in dogs are ataxia and hypersensitivity to external stimuli. Vomiting and hypersalivation, sustained muscle activity which may result in secondary hyperthermia, and seizures have also been observed. In extreme cases intoxication may lead to death (Hayes et al. 1976). PTA is rapidly absorbed following exposure, as evidenced by the acute onset of clinical signs, although quantitative absorption data are lacking. After systemic uptake, PTA has been detected in the liver, kidneys, brain, serum, and gastrointestinal tract of exposed animals. Additionally, several phase I metabolites have been found in vitro (Eriksen et al. 2013; Uhlig et al. 2020).

PTA and other tremorgenic indole diterpene mycotoxins cause multiple disruptions in both the central and peripheral nervous system (for a review, see Eriksen et al. 2013). PTA antagonizes high-conductance Ca^2+^-activated potassium channels (BK channels) in smooth muscles and peripheral tissues. In the central nervous system, PTA increases the spontaneous presynaptic release of the excitatory neurotransmitters glutamate and aspartate and the inhibitory neurotransmitter GABA from cerebrocortical synaptosomes. Further, PTA causes oxidative stress which may be a pathophysiological mediator in neurotoxicity (Berntsen et al. 2013; Eriksen et al. 2013).

Diagnostic confirmation of tremorgenic mycotoxicosis in veterinary practice remains challenging, in part because of the lack of simple routine methods. All studies published so far used chromatographic methods, in recent years exclusively liquid chromatography with tandem mass spectrometry (LC-MS/MS, Eriksen et al. 2013; Uhlig et al. 2020). Rapid on-site tests, or enzyme immunoassay (EIA) methods, which would be helpful under the conditions of veterinary practice, are not commercially available for this purpose.

In this situation, it is no surprise that only sporadic case reports exist across North America (Richard and Arp 1979; Richard et al. 1981; Boysen et al. 2002; Walter 2002; Young et al. 2003), Australia and New Zealand (Hocking et al. 1988; Munday et al. 2011), South Africa (Naudé et al. 2002) and Europe (Eriksen et al. 2010; Uhlig et al. 2020; Novotna et al. 2023). Given the widespread occurrence of *P. crustosum* from arctic to tropical regions (Sonjak et al. 2005), and considering the high frequency of this fungus in household and outdoor settings, it is likely that cases are underdiagnosed. This underlines the need for more systematic surveillance and analytical confirmation.

The aim of this study was to combine clinical data from cases of acute tremorgenic poisoning events presented at the veterinary clinic of Justus-Liebig University with analytical data for mycotoxins in gastric content, in fungal cultures from gastric content, and in blood serum of intoxicated dogs. Furthermore, the use of EIAs for several groups of tremorgenic mycotoxins should be assessed, with the intention to establish the requirements of a complimentary diagnostic tool for point-of-care testing for the most relevant compounds.

## Materials and methods

### Chemicals and reagents

For the enzyme immunoassay analyses, penitrem A (PTA; molecular weight: 634.21 g/mol) was purchased from Cayman Chemical Company (Ann Arbor, USA) and dissolved in methanol to obtain a concentration of 1 mg/mL. Paxilline (PAX; molecular weight 435.6 g/mol) was purchased from ENZO Life Sciences (Lörrach, Germany). Cyclopiazonic acid (CPA); molecular weight 336.38 g/mol) and ergometrine (ergonovine) were from Sigma Aldrich (Taufkirchen, Germany). All mycotoxins were purchased in crystalline form. The concentrations of the toxin standard solutions were checked by UV-spectroscopy. Casein sodium salt, 3,3´,5,5´-tetramethyl benzidine (TMB) and Tween 20 were also purchased from Sigma-Aldrich. Swine anti-rabbit IgG-HRP was from DAKO (Hamburg, Germany). All other reagents used were at least of analytical grade, methanol for dissolving mycotoxin standards was spectroscopy grade. Malt extract agar (MEA), potato extract agar and Sabouraud dextrose agar was obtained from Oxoid Limited (Hampshire, United Kingdom). EIA microtiter plates were from Thermo Fisher Scientific (Waltham, USA). Solid phase antigens for plate coating were diluted with sodium bicarbonate buffer (0.05 mol/L, pH 9.6). Blocking solution was 2% casein sodium salt in phosphate-buffered saline (PBS, 0.01 mol/L, pH 7.2), plate wash solution was distilled water containing 0.25 mL Tween 20 and 8.5 g NaCl per litre. The toxin standard solutions were prepared in 10% methanol/PBS. Swine anti-rabbit IgG-HRP was prepared with 1% casein sodium salt in PBS. Enzyme substrate/chromogen solution was prepared according to Ackermann et al. (2011).

Acetonitrile (LC-MS grade) was purchased from Fisher Scientific (Schwerdte, Germany). Water ASTM type I (> 18 MΩ) was produced with a Purelab Flex system (ELGA, Bielefeld, Germany). Formic acid was purchased from Merck KGaA (Darmstadt, Germany). For the mass spectrometric analyses, penitrems A-F were isolated from *P. crustosum* as previously described (Kalinina et al. 2017), whereas PAX and CPA were purchased from Enzo Life Sciences.

### Patients and sample material

All five dogs had been presented at the clinic for small animals of Justus-Liebig-University (October 2022-June 2023), Giessen, Germany, because of rapid onset of acute tremor and other neurological symptoms. Details on these patients, including clinical findings and specifics on therapeutic treatment, are summarized in Table 1. Vomiting, which is often associated with tremorgenic mycotoxicosis (Naude et al., 2002; Eriksen et al. 2013), was reported by the owner only for dog 4. All dogs had, according to statements made by the owners, ingested foreign material shortly before onset of clinical symptoms which were dominated by tremor. Treatment in all cases included non-specific detoxification therapy, either gastric lavage (dog 1) or induced emesis (dogs 2–5), oral application of activated charcoal, intravenous fluid therapy with balanced isotonic crystalloid solution and lipid therapy. Depending on the severity of clinical symptoms, further symptomatic treatment was performed (Table 1). Gastric content, either material from gastric lavage (dog 1) or vomit from induced emesis (dogs 2–5) was secured for further analysis. Small amounts of leftover blood serum from laboratory analysis were available for mycotoxin analysis from dogs 3–5.

**Table 1:** Summary of case characteristics, anamnesis, clinical symptoms, therapy, and outcome of five dogs with tremorgenic mycotoxicosis

	Dog 1 (October 2022)	Dog 2 (April 2023)	Dog 3 (April 2023)	Dog 4 (May 2023)	Dog 5 (June 2023)
Breed, sex	Labrador, ♀	Australian shepherd, ♂ neutered	Mixed breed, ♀	Mixed breed, ♂ neutered	Eurasian, ♀
Age (years)	6	3	3	12	< 1
Body weight (kg)	28.4	31	30	25	21
Preliminary information on presentation at clinic	Unknown material ingested during walk, onset of tremor approx. 30 min after ingestion	Material from organic garbage can ingested, after 2 h onset of generalized tremor	Bread from horse stable environment ingested, acute onset of symptoms after a few minutes	Unknown material ingested during walk, after 30 min acute onset of generalized tremor, lateral recumbent position, emesis	Unknown material ingested at home, just before acute onset of tremor
Main symptoms	Muscle tremor, hypersalivation, tachypnea, tachycardia	Generalized moderate tremor, tachypnea, tachycardia	Gait instability, tremor, hyperexcitability	Generalized tremor, vestibular/cerebellar ataxia, right-sided strabismus, apathy	Generalized muscle tremor, cerebellar ataxia, hypersensitivity to auditory and tactile stimuli
Additional symptoms observed	Restlessness, anxiousness, disorientation, apathy	Hyperesthesia, hyperekplexia, generalized vestibular/cerebellar ataxia, hypermetria of all limbs, mild bilateral mydriasis	Cerebellar ataxia, intention tremor, apathy, hyperthermia, diminished consciousness, hyperexcitability to auditory, visual, and tactile stimuli, increased muscle tone in extremities	Reduced consciousness, hyperexcitability to auditory, visual, and tactile stimuli, hypersalivation	-
Breath rate	48/min	60/min	24/min	28/min	-
Pulse rate	184/min	136/min	160/min	112/min	-
Body temperature	38.2 °C	38.8 °C	41.7 °C	37.9 °C	-
Immediate treatment	Anesthesia with ketamine, propofol and midazolam, gastric lavage	Induced emesis with apomorphine hydrochloride solution (0.08 mg/kg), anticonvulsant therapy with levetiracetam (60 mg/kg) and diazepam (10 mg rectal tube)	Induced emesis with apomorphine hydrochloride solution (0.08 mg/kg), cooling, anticonvulsant therapy with diazepam (0.5 mg/kg), levetiracetam (20 mg/kg), phenobarbital (2.5 mg/kg), midazolam (0.2 mg/kg/h), methocarbamol (50 mg/kg) as muscle relaxant	Induced emesis with apomorphine hydrochloride solution (0.08 mg/kg), anticonvulsant therapy with diazepam (0.5 mg/kg) and methocarbamol (40 mg/kg) as muscle relaxant	Induced emesis with apomorphine hydrochloride solution (0.08 mg/kg), anticonvulsant therapy with midazolam, methocarbamol as muscle relaxant
Gastric lavage/vomit	Gastric lavage containing solids resembling compost material	Vomit	Vomit containing organic and inorganic waste material	Vomit	Vomit
Detoxification therapy	Activated charcoal (0.5 g/kg), fluid therapy with balanced isotonic crystalloids (120 mL/h), lipid therapy (one time 210 mL 20% lipid emulsion within 60 min slowly i.v., after that 4x daily 60 ml lipid emulsion bolus within 15 min slowly i.v.), antiemetic therapy (1 mg /kg maropitant)	Activated charcoal, fluid therapy with balanced isotonic crystalloids (2 mL/kg/h), lipid therapy, antiemetic therapy	Activated charcoal, fluid therapy with balanced isotonic crystalloids (6 mL/kg/h), lipid therapy (4x daily 48 ml within 15 min i.v.), antiemetic therapy	Activated charcoal, fluid therapy with balanced isotonic crystalloids (4 mL/kg/h), lipid therapy (4x daily 50 mL within 15 min i.v.), antiemetic therapy	Activated charcoal, fluid therapy with balanced isotonic crystalloids, antiemetic therapy
Outcome	Discharged free of symptoms after 2 days	Free of symptoms after 48 h, discharged after 3 days	Discharged for treatment at home after 4 days with mild symptoms of cerebellar ataxia and tremor	Discharged free of symptoms after 5 days	Discharged free of symptoms within 1 day

### Sample preparation for gastric material

One gram of liquid gastric material (containing variable amounts of small solid particles) was thoroughly mixed with 10 mL methanol/water (70/30, *v/v*) in a 50 mL polypropylene (PP) screw cap tube on a wrist-action shaker for 5 min with short intervals. The solids were removed by centrifugation at 3000 × *g* for 15 min. The supernatant was transferred into a 14 mL PP tube. For EIA analysis, a portion of the supernatant was diluted at least 1:7 with PBS pH 7.2. Further dilutions were made according to the respective EIA protocol.

### Isolation and identification of fungi from gastric content

Using a sterile swab (pre-moistened with sterile distilled water), gastric material from each dog was transferred onto malt extract agar, potato extract agar and Sabouraud dextrose agar supplemented with chloramphenicol using the three-point inoculation method. After 5 days at 25 °C, fungal growth was observed on all plates. Colonies appeared greyish-green blue with a light yellow or white border. Following primary isolation, representative fungal colonies were subjected to single-colony purification. Well-separated colonies were selected and transferred on to fresh malt extract agar plates. This purification procedure was repeated up to two times until morphologically uniform cultures were obtained, as confirmed by microscopic examination. From each plate, a 1 × 1 cm square of agar showing dense fungal growth was cut out, extracted with 1 mL of methanol, centrifuged, and further diluted at least 1:10 with PBS for EIA analysis as described previously (Rehagel et al. 2022).

Further identification of the isolates at the species level was performed by sequence analysis of the internal transcribed spacer (ITS) region and a partial fragment of the β-tubulin gene (*benA*), as described by Guevara-Suarez et al. (2016). Genomic DNA was extracted from fresh mycelium harvested from pure colonies grown on malt extract agar plates using DNeasy Blood and Tissue kit (Qiagen, Hilden, Germany) following the manufacturer’s instructions. Amplification of the ITS region and the β-tubulin gene fragment was carried out using the primer pairs ITS4/ITS5 (White et al. 1990) and Bt2a/Bt2b (Glass and Donaldson 1995), respectively, according to published protocols. The PCR products were purified using the QIAquick PCR Purification Kit (Qiagen) following the manufacturer’s recommendations, and sequencing was performed by SeqLab (Göttingen, Germany). The obtained DNA sequences were compared with reference sequences using the Basic Local Alignment Search Tool (BLAST) at the National Center for Biotechnology Information (NCBI).

### EIA analysis of PTA, PAX, CPA, and ergoline alkaloids

All EIAs were in-house methods developed previously in our lab, including tests for PTA (Rehagel et al. 2022), PAX (Bauer et al. 2017), CPA (Hart et al. 2026), and ergoline alkaloids (Gross et al. 2018). Characteristics of these test systems are summarized in Online Resource 1, Table 1. With the exception of ergoline alkaloids, limited information is available on test specificity because of the lack of relevant structurally similar toxin analogues. Furthermore, the toxin composition in gastric samples was unknown. Therefore, all EIA results were regarded as qualitative screening tests, although measurements were done on the basis of the standard curves for the reference toxin in each test system. The concentration resulting in 20% binding inhibition compared with the negative control solution (blank) was used as a cut-off value to establish lower detection limits (LOD). The upper limit of the dynamic detection range was set at the concentration resulting in 80% binding inhibition. Calculated from the standard curve detection limit, multiplied with minimum sample dilution factor (liquid gastric content: 70; agar plugs: 10), detection limits for PTA, PAX, CPA and ergoline alkaloids in samples were at 6.3 µg/mL, 0.036 µg/mL, 2.3 µg/mL, and 0.0017 µg/mL in gastric material. The detection limit for PTA, CPA and ergoline alkaloids in fungal culture material (µg/cm^2^ agar plug) were at 0.9 µg/mL, 23 ng/mL, and 0.24 ng/mL. PAX was not determined in agar plug extracts by EIA. All analyses were performed in quadruplicate wells and in at least three dilutions per extract. For estimation of the toxin content, expressed as equivalents to the standard curve reference toxin, the mean result for all dilutions yielding absorbance values within the detection range was used. If all dilutions yielded absorbance values exceeding the upper dynamic range, extracts were reanalysed at higher dilutions as appropriate.

### HPLC-MS/MS analysis

For sensitive analysis and (semi-)quantification of penitrems and PAX in gastric content and serum samples, a UHPLC-MS/MS system was used. A 1260 Infinity II binary LC system (Agilent, Waldbronn, Germany) was coupled to a QTRAP 5500 mass spectrometer (Sciex, Darmstadt, Germany). For a preliminary overview of present analytes, 10 µL of gastric lavage extract were diluted in 90 or 490 µL of water/acetonitrile (70/30, *v/v*) containing 0.1% formic acid (*v/v*), respectively. Protein precipitation of serum samples was done by adding 40 µL of acetonitrile to 10 µL of serum and strong shaking, followed by addition of 50 µL water containing 0.1% formic acid (*v/v*). All sample types were then centrifuged at 15,000 × *g*, at room temperature for 10 min and the supernatant transferred to sample vials for injection of 20 µL to the LC-MS/MS system. Chromatographic separation was done on a ReproSil-Pur 120 C18-AQ column (2.0 × 150 mm, 3 μm) equipped with a guard column of the same material (2.0 × 5.0 mm) (both Dr. Maisch HPLC GmbH, Ammerbuch, Germany). The following linear gradient of water (A) and acetonitrile (B) both containing 0.1% formic acid was used at a flow rate of 450 µL/min: 0 min 30% B, 0.5 min 30% B, 8 min 100% B, 11 min 100% B, 11.1 min 30% B, 15 min 30% B. Column temperature was held at 40 °C. Ionization was done with an electrospray in positive ionization mode at a voltage of 5500 V and temperature of 400 °C. Curtain gas, nebulizer gas (gas 1) and drying gas (gas 2) were set to 30 psi, 35 psi and 45 psi, respectively. The gas used for collision fragmentation was set to an instrument value of 2 arbitrary units. For each compound one MRM transition was used for quantification, while one or two more were monitored for qualification. More detailed information on the MRM parameters is given in Online Resource 1, Table S2. Data acquisition was done with Analyst Software 1.6.2, while data analysis was done using Sciex OS 3.1 software.

For the gastric lavage extract samples, with quantifiable amounts of penitrems and PAX, standard addition according to AQC-Guidelines was performed. Four addition levels ranging from 0.5- to 2-times the expected amount in the samples were used to calculate the amount in the native sample. Penitrems B-F and PAX were semi-quantified by their similar signal responses compared to PTA. CPA was only measured qualitatively.

## Results

Five dogs of various breeds (Labrador, Australian Shepherd, Eurasian, and mixed breeds), aged < 1 to 12 years and weighing 21–31 kg, were presented with acute onset of neurological signs consistent with tremorgenic mycotoxicosis following suspected ingestion of unknown or organic waste material (Table 1). In all cases, clinical signs developed rapidly (within minutes to 2 h) and were dominated by generalized tremors, frequently accompanied by tachypnea, tachycardia, and varying degrees of cerebellar or vestibular ataxia. Additional neurological abnormalities included hyperexcitability, hyperesthesia, altered consciousness, and, in severe cases, hyperthermia and recumbency.

Initial management consisted of prompt decontamination, including induction of emesis or gastric lavage, combined with anticonvulsant and muscle relaxant therapy. All dogs received activated charcoal and intravenous fluid therapy, while four of five cases were additionally treated with intravenous lipid therapy. Clinical recovery was favorable in all cases, with four dogs discharged without residual clinical signs within 1–5 days and one dog discharged with mild transient neurological deficits after 4 days.

EIA analysis of gastric content from all five dogs yielded highly positive results (PTA equivalents) in a range from 81 µg/ml to 480 µg/ml (Table 2). EIA analysis of gastric content from dog 1 also gave highly positive results for PAX, but due to problems with test performance, no quantitative result could be obtained. Therefore, the PAX EIA was discontinued in further analysis. In addition to PTA, gastric content was positive in all cases for ergoline alkaloids, and in case of dogs 4 and 5 also for CPA, although at lower levels than for PTA.

**Table 2:** EIA results for PTA (penitrem A-equivalents), PAX, CPA, and ergoline alkaloids in gastric content [µg/mL], and in *P. crustosum* culture material (malt extract agar) isolated from gastric content [µg/cm^2^ agar plug]

Dog #	Sample material	Test system for			
		PTA	PAX	CPA	Ergoline alkaloids
1	Gastric content	300	+	< 1.6	0.004
	*P. crustosum* **SC1-1-22** culture material	18	n.a.	< 0.23	< 0.24
2	Gastric content	480	n.a.	< 1.6	0.005
	*P. crustosum* **SC3-1-23** culture material	44	n.a.	< 0.23	< 0.24
3	Gastric contentl	390	n.a.	< 1.6	0.010
	*P. crustosum* **SC4-1-23** culture material	25	n.a.	< 0.23	< 0.24
4	Gastric content	81	n.a.	2.7	0.010
	*P. crustosum* **SC6-1-23** culture material	26	n.a.	< 0.23	< 0.24
5	Gastric content	110	n.a.	2.9	0.030
	*P. crustosum* **SC7-1-23** culture material	26	n.a.	< 0.23	< 0.24

*n*.*a* not analysed

Gastric content was then analysed by LC-MS/MS (Table 3). Penitrems A-F in varying ratios were detected in all samples, the toxin cocktail dominated by either PTA (dogs 1, 5) or by penitrem E (dogs 2–4). The total penitrem concentration estimated from LC-MS/MS results varied from 0.079 µg/ml to 9.57 µg/ml. PAX was found in three samples (dogs 1, 4, 5), at levels of 0.011 µg/ml to 0.2 µg/ml. CPA was not determined in sample 1, but traces were found in samples 2–5. Ergoline alkaloids were not analysed by LC-MS/MS.

**Table 3:** HPLC-MS/MS analysis results for penitrems, PAX, and CPA in gastric content and in serum [ng/mL]

Dog No./ analysed material	PTA	PTB	PTC	PTD	PTE	PTF	Sum of PTA-PTF	PAX	CPA
1/ gastric content	7,190	880	200	880	270	150	9,570	180	n.a.
2/ gastric content	131	92.5	10.4	74.7	253	n.d.	562	< LOQ	< LOQ
3/ gastric content	65.4	9.2	< LOQ	6.6	69.8	n.d.	151	n.d.	n.d.
3/ serum	< LOQ	< LOQ	n.d.	n.d.	n.d.	n.d.	Positive	n.d.	n.d.
4/ gastric content	162	48.4	1.4	33.9	216	2.0	464	11.5	< LOQ
4/ serum	n.d.	< LOQ	n.d.	n.d.	n.d.	n.d.	Positive	n.d.	< LOQ
5/ gastric content	64.1	5.9	0.7	2.9	5.6	< LOQ	79.2	201	< LOQ
5/ serum	n.d.	n.d.	n.d.	n.d.	n.d.	n.d.	Negative	< LOQ	n.d.

*n*.*d* none detected, *n*.*a* not analysed

Blood serum (dogs 3–5) was analysed by LC/MS-MS only, because the sensitivity of the EIA methods was considered to be insufficient. Traces (< LOQ) of PTA (dog 3) and PTB (dogs 3, 4) were found in two samples. Serum from dog 5, from which the lowest result for penitrems, and highest result for PAX, in gastric content had been obtained, was negative for PTA-PTF, but contained detectable levels of PAX. Only serum from dog 4 was positive for CPA by LC/MS-MS. Gastric content from this dog also had the highest level of CPA, as determined by EIA.

Fungal growth was obtained for gastric material from all five dogs, on malt extract agar, potato extract agar and Sabouraud dextrose agar supplemented with chloramphenicol. Gastric content from dogs 1–3 yielded uniform pure culture of similar macroscopic appearance (greyish-green blue with a light yellow or white border). For dogs 4 and 5, this colony type was also dominant on all plates, but mixed colony growth was observed. Based on microscopic characteristics, the isolates were preliminarily identified as *Penicillium* spp. Further analysis of isolates by using sequence analysis of the ITS and beta-tubulin genes unequivocally showed the presence of *P. crustosum* in all five cases (Table 4). For dogs 4 and 5, two other species (*Mucor racemosus*,* Geotrichum silvicola*) could be isolated from gastric content. When agar plugs from all isolates were tested in the PTA EIA, all *P. crustosum* isolates were found to be highly positive (18–44 µg/cm^2^ agar plug; Table 2). In contrast, all isolates were negative for CPA and ergoline alkaloids. The isolates of *Mucor racemosus* and *Geotrichum silvicola* were also tested, but were negative in all EIA systems.

**Table 4:** Identification of fungal isolates from gastric content of five dogs based on sequence analysis of the internal transcribed spacer (ITS) region and partial beta-tubulin gene. All isolates had originally been isolated from Sabouraud dextrose agar

Dog #	No. of isolates	Designation of isolate	Phylogenetic affiliation	Gene analysed	Sequence length (bp)	Identity % (Query Coverage)	Closest species (NCBI accession number)
1	1	**SC1-1-22**	***P. crustosum***	ITS region	577	100 (100)	*P. crustosum* strain FRR 1669 (NR_077153.1)
				beta-tubulin	374	100 (100)	*P. crustosum* strain NRRL 66,388 (KY172962.1)
2	1	**SC3-1-23**	***P. crustosum***	ITS region	576	100 (100)	*P. crustosum* strain FRR 1669 (NR_077153.1)
				beta-tubulin	441	99.77 (100)	*P. crustosum* strain NRRL 66,388 (KY172962.1)
3	2	**SC4-1-23**	***P. crustosum***	ITS region	589	100 (99)	*P. crustosum* strain FRR 1669 (NR_077153.1)
				beta-tubulin	437	99.31 (100)	*P. crustosum* strain CMV017D2 (MN031393.1)
		SC4-2-23	*Mucor hiemalis*	ITS region	1166	99.85 (99)	*Mucor hiemalis* strain W2R2 (MT084004.1)
				beta-tubulin	-	-	No specific amplicon obtained
4	2	**SC6-1-23**	***P. crustosum***	ITS region	578	100 (99)	*P. crustosum* strain FRR 1669 (NR_077153.1)
				beta-tubulin	428	100 (99)	*P. crustosum* strain CMV011E5 (MK451206.1)
		SC6-2-23	*Mucor racemosus*	ITS region	560	100 (95)	*Mucor racemosus f. racemosus* CBS 260.68 (NR_126135.1)
				beta-tubulin	-	-	No specific amplicon obtained
5	3	**SC7-1-23**	***P. crustosum***	ITS region	567	99.82 (99)	*P. crustosum* strain FRR 1669 (NR_077153.1)
				beta-tubulin	431	100 (99)	*P. crustosum* strain CMV011E5 (MK451206.1)
		SC7-2-23	*Mucor racemosus*	ITS region	620	99.83 (93)	*Mucor racemosus f. racemosus* CBS 260.68 (NR_126135.1)
				beta-tubulin	-	-	No specific amplicon obtained
		SC7-3-23	*Geotrichum silvicola*	ITS region	385	99.04 (80)	*Geotrichum silvicola* strain CBS 9194 (NR_077071.1)
				beta-tubulin	-	-	No specific amplicon

## Discussion

Canine tremorgenic toxicosis appears to be a quite frequent occurrence in Germany, with often dramatic acute clinical pictures, but in most cases with non-lethal outcome. Videos demonstrating clinical symptoms for dogs 3–5 from our study are added as supplement material (Online Resources 2a, 2b,2c). These videos were recorded after treatment had begun, after the most severe symptoms of intoxication had subsided. Yet, they demonstrate all signs of tremorgenic mycotoxin poisoning, generalized tremor and ataxia. The fact that all five dogs recovered within 1–5 days under therapy seems to be a general feature of this type of intoxication (Kearley et al. 2024), although in some cases neurological symptoms may persist for years (Eriksen et al. 2010). A relatively high number of suspected cases of poisoning of dogs with tremorgenic mycotoxins has been reported in recent years from Germany by Fritz et al. (2020) and Braun et al. (2024), mostly associated with the ingestion of moldy walnuts, but none of these studies had included analyses of mycotoxins. On a global scale, sporadic reports of suspected tremorgenic mycotoxicosis in dogs have been published from many regions of the world over the last decades (Richard and Arp 1979; Hocking et al. 1988; Boysen et al. 2002; Munday et al. 2011; Novotna et al. 2023), but few studies provide sufficient analytical evidence for canine tremorgenic mycotoxicosis (Eriksen et al. 2010).

In this study, we were able to combine five clinical cases with mycological analyses and mycotoxin analyses. Preliminary information provided by the dog owners all included the fact that unknown material was ingested by the animals during walk or at home. Clinical symptoms in all cases were typical for tremorgenic mycotoxicosis, although the symptoms are also resembled by those observed after ingestion of rodenticides containing α-chloralose (Adamik and Sigrist 2009; Dijkman et al. 2022). Fortunately, all five dogs included in this study could be discharged from the clinic after 1–5 days of symptomatic treatment, either completely asymptomatic or with just minor symptoms which could further be treated at home.

We considered it important to confirm that tremorgenic mycotoxins had indeed been the cause of toxicosis, for two reasons: first, identification of the cause of intoxication may be helpful for therapy and support estimates of prognosis. Second, it would also allow discrimination between intoxication with “natural toxins” and “man-made agents”. The latter agents include insecticides, rodenticides and molluscicides (Bertero et al. 2020; De Roma et al. 2017; Dutil and Berny 2023). Even compounds like strychnine, which has been banned within the European Union since 2006, was still found to be a major cause of tremorgenic poisoning in dogs in Italy (Bertero et al. 2020). To our knowlegde, α-chloralose in poisoned bait appears to be a major problem in Germany, either set for rodent control, or intentionally spread to kill dogs (Adamik and Sigrist 2009; Dijkman et al. 2022). The latter could also have legal implications in aspects of criminal liability.

In this study, we were able to record clinical data (Table 1) and combine them with analytical findings for tremorgenic fungi (Table 4), mycotoxins in gastric content (Tables 2 and 3), and in three cases information on mycotoxins in blood serum could be obtained (Table 3). Both after screening by EIA and by subsequent analysis using LC-MS/MS, penitrems, PAX, and CPA were found in gastric content material. At least in case of the penitrems, the levels were high enough to explain the clinical symptoms. The minimum tremorgenic dose for PTA in dogs is at 0.5 mg/kg body weight, as reported by Hayes et al. (1976), and PTA was probably the most potent tremorgen among the toxins under study. We therefore assume that in acute clinical cases, the toxin concentration in the stomach, shortly after ingestion, had to be much higher than 1 µg/ml, at a breed-dependent maximum total stomach volume of 0.5–8 L (Deschamps et al. 2022). Although the total amount of ingested penitrems by each dog could not be determined, the concentrations found in gastric content material from vomit or gastric lavage suggest that the total amount of toxins ingested must have been in the milligram range, sufficient to evoke tremor and other symptoms (Eriksen et al. 2013). The levels of penitrems found in our study in the microgram per gram range are largely in agreement with those reported by others (Naudé et al. 2002; Kearley et al. 2024) The unique mixture of potent neurotoxic mycotoxins in gastric content as found in our study, including penitrems A-F, PAX, CPA, and ergoline alkaloids has not been reported previously. While these toxins all belong to the large family of indole alkaloids, PTA, PAX, and CPA primarily act trough effects on potassium channels, causing tremors (Reddy et al. 2019), some ergoline alkaloids may also specifically affect the central nervous system. Unfortunately, the exact compounds could not be determined by the group specific EIA, and no suitable chromatographic method was available in this study. However, it seems reasonable to assume that such a neurotoxic cocktail may exert synergistic effects. Further work should include a detailed analysis of ergolines, and possibly include other compounds such as roquefortine C which has been associated with tremors in dogs in previous studies (Naudé et al. 2002; Eriksen et al. 2010; Novotna et al. 2023). The impact of co-ingestion of multiple neurotoxic mycotoxins has yet to be elucidated.

Blood serum from dogs 3–5 was available for analysis by LC-MS/MS. All three samples contained at least trace amounts of penitrems. These findings are remarkable, since it demonstrates that these three toxins actually had been absorbed and entered the circulatory system. For PTA, the only other report is that of Uhlig et al. (2020). These authors also qualitatively identified trace amounts of PTA and PTA metabolites in one out of four blood samples from intoxicated dogs. To our knowledge, evidence for PAX and CPA in blood serum from intoxicated dogs has not been reported previously. In our cases 3–5, the blood samples collected for determination of clinical chemistry parameters had been taken several hours after ingestion of toxins, therefore no information about the kinetics of absorption and distribution of the toxins could be obtained. However, the fact that mycotoxin poisoning of dogs may include a cocktail of penitrems, PAX and CPA indicate the necessity for further studies.

Further evidence that *P. crustosum* was at least the dominant fungal species responsible for poisoning, and for PTA productivity of these isolates, was obtained from mycological analyses of gastric content, followed by PTA determination in fungal culture by EIA. *P. crustosum* was the only fungal species which could be isolated from gastric content of dogs 1 and 2, and was the dominant species in all other gastric samples. Gastric samples of dogs 3–5 additionally yielded saprophytic *Mucor* species or yeast (*Geotrichum silvicola*). All isolates of *P. crustosum* were found to be potent producers of PTA equivalents by EIA (18–44 µg/cm^2^ agar plug), while the other fungal isolates were negative. Comparison of EIA results with that obtained by LC-MS/MS showed qualitative agreement, but quantitatively the EIA concentrations greatly exceeded the LC-MS/MS values, most notably for the penitrems. Matrix effects were considered unlikely because of the high sample dilutions (> 1:500) necessary to reach the measuring range of the standard curve. The overestimation could possibly be explained by strong relative cross-reactivity (> 100%) of some of the structural analogues penitrem B (PTB) - penitrem F (PTF) in the PTA EIA and their overall high contribution to the total penitrem content. According to the comparison of EIA results with LC-MS/MS results for gastric content, it is reasonable to assume that several cross-reactive penitrems have contributed to these results, and that these results may represent an overestimate of the sum of penitrems A-F, but unfortunately the composition of toxins could not be elucidated in this study. However, an extremely high PTA level of 31 milligram per culture medium inoculated with a *Penicillium* isolate from gastric content of a dog with suspected mycotoxicosis has recently been reported by Novotna et al. (2023), although details on the size or volume of the analysed medium has not been mentioned by the authors. In “rice vomit” of two dogs with suspected tremorgenic mycotoxicosis, in a case of acute toxicosis, pure culture of *P. crustosum* in gastric content seems to be not unusual, as reported by Naudé et al. (2002). Naudé (2002) also found pure culture of *P. crustosum* in a dog with tremorgenic intoxication. Some older studies, which had focused on mycological analysis of feed material suspected as the source of intoxication and toxin production, all found PTA producing *P. crustosum* (Richard and Arp 1979; Richard et al. 1981; Hocking et al. 1988; Munday et al. 2011).

Some limitations of the study should be acknowledged. The total amount of toxins ingested via contaminated material remains unknown, because the amount of vomit or gastric lavage liquid was highly variable and a direct relationship between the toxin concentration and intestinal toxin resorption could not be established. It has been suggested that bioavailability of PTA is high, which would fit with observed early onset of clinical signs in dogs (Uhlig et al. 2020). If we consider toxin levels in the microgram/mL range in the gastric fluid samples and assume that a high percentage of the toxins had already been absorbed at the time of induced emesis, we may speculate that each of the dogs may have ingested PTA and other tremorgens in milligram quantities. This is further supported by the fact that in culture, all *P. crustosum* isolates from vomit or gastric lavage were very strong producers of PTA. The co-occurrence of PTA, PAX, CPA, and ergoline alkaloids in gastric samples from which only *P. crustosum* as a toxinogenic species was identified raises the question about a possible unidentified fungal species. *P. crustosum* so far has not been reported as an established producer of PAX, CPA, and ergoline alkaloids. Further work should be directed at the mycotoxin profile and the possibility of presence of toxigenic fungal species which are not culturable on the media used in this study. Finally, because this was an observational study and not an invasive study, we had to rely on waste or leftover material. Therefore, several aspects such as toxicokinetics and toxin elimination could not be studied.

## Conclusions

To the best of our knowledge, this work presents the most complete set of analytical data in tremorgenic mycotoxicosis so far. For five clinical cases of canine tremorgenic poisoning, information of tremorgenic mycotoxins in gastric content and blood serum are presented, combined with mycological assessment of gastric content and analysis of toxin production by fungal isolates from gastric content. In conclusion, we could demonstrate, without reasonable doubt, that indeed tremorgenic mycotoxins were the cause of clinical symptoms. Tremorgenic mycotoxicosis should be considered an important differential diagnosis in dogs presenting with acute-onset generalized tremors, particularly when access to mouldy food, feed, waste, or compost is suspected. This may be important if intentional deeds by “dog-haters” with, for example, poison baits containing α-chloralose or other rodent poisons, is suspected by the dog owners. While diagnostic confirmation can be achieved through toxicological and mycological analyses, initiation of therapy should not be delayed. EIA represents a rapid, cost-effective, and reliable screening tool for the detection of mycotoxins in gastric contents and serum. Mycological culture, although more time-consuming and irrelevant for routine veterinary practice, can provide complementary evidence in special cases having forensic relevance, as *P. crustosum* was consistently isolated in the present cases, and all isolates in this study were toxigenic. Early implementation of detoxification measures in combination with symptomatic treatment, including anticonvulsants, muscle relaxants and lipid therapy, is associated with an excellent prognosis. Due to their high lipophilicity, penitrems are likely responsive to lipid-based therapy, supporting its recommendation in affected patients and underscoring the importance of toxin identification, as such approaches are ineffective for less lipophilic compounds like α-chloralose. Rapid diagnosis of mycotoxins would provide very helpful information for therapy and prognosis, and could exclude other causes of intoxication, for example intentionally set poison baits. The EIAs used in this study present a first step towards rapid point of care lateral flow testing systems. 

## Supplementary Information

Below is the link to the electronic supplementary material.

Supplementary Material 1

Supplementary Material 2

Supplementary Material 3

Supplementary Material 4

## Data Availability

No datasets were generated or analysed during the current study.
