# Supplementary material for: Five cases of tremorgenic mycotoxicosis in dogs – clinical aspects and analytical methods
Source: Vet Res Commun. 2026 Aug 11;50(5):517. doi: 10.1007/s11259-026-11458-z (PMC13461703; doi:10.1007/s11259-026-11458-z)
Supplement: Supplementary file 1 [file 11259_2026_11458_MOESM4_ESM.pdf]

Five cases of tremorgenic mycotoxicosis in dogs – clinical aspects and analytical methods

Veterinary Research Communications

Leon Hart*, Christina Rehagel, Michael Kuhn, André Taube, Lukas Jozefowitz, Esther Hassdenteufel, Benedikt Cramer, Ömer Akineden, Ewald Usleber

*Corresponding author: Chair of Dairy Sciences, Institute of Veterinary Food Science, Justus-Liebig-University Giessen, Germany; Leon.Hart@uni-giessen.de

Online resource 1

Table S1 and Table S2

Table S1. Overview of characteristics of the enzyme immunoassays (EIA) used in this study

Test system	Standard curve		Calculated detection limit in		Specificity	Reference
	Toxin	Detection limit, ng/ml*	Liquid gastric material (µg/ml)**	Fungal culture µg/cm^2 agar plug***		
Penitrem A (PTA)	PTA	90±21	6.3	0.9	No cross-reaction with PAX, lolitrem B, terpendole E, verruculogen; possibly reacts with penitrems B-F	Rehagel et al., 2022
Paxilline (PAX)	PAX	0.52±0.02	0.036	n.a.	13-desoxypaxilline, possibly other PAX analogues	Bauer et al., 2017
α-Cyclopiazonic acid (CPA)	CPA	2.3±1.2	1.6	0.23	Acetyl-CPA	Hart et al., 2026
Ergoline alkaloids	Ergometrine	0.024±0.005	0.0017	0.24	Ergot and clavine alkaloids	Gross et al. 2018

*Cut-off set at 20% binding inhibition relative to the absorbance of the blank; values represent the mean and standard deviation of 5 standard curves each.
**Minimum sample dilution factor: 70; Minimum sample dilution factor:10. n.a., not applicable.

Table S2. MS parameter for MRM detection used for the analysis of penitrems A-F, paxilline and cyclopiazonic acid. Transitions in bold used as quantifier. Entrance potential of 10 V and collision cell exit potential of 11 V used for all transitions. CE: collision energy, DP: declustering potential.

Analyte	Q1 mass [*m/z*]	Q3 mass [*m/z*]	Dwell time [ms]	CE [V]	DP [V]
**Penitrem A**	**634.3**	**616.2**	**20**	**15**	**150**
	634.3	558.5	20	25	150
	634.3	540.0	10	33	150
**Penitrem B**	**584.5**	**566.3**	**20**	**24**	**150**
	584.5	514.4	20	31	150
	584.5	497.4	10	30	150
**Penitrem C**	**602.3**	**532.4**	**20**	**28**	**130**
	602.3	584.2	20	20	150
	602.3	532.4	20	28	130
**Penitrem D**	**568.3**	**550.3**	**20**	**25**	**140**
	568.3	481.3	20	30	140
	568.3	498.6	20	25	140
**Penitrem E**	**600.2**	**524.4**	**20**	**25**	**80**
	600.2	582.4	20	19	80
	600.2	506.0	10	36	80
**Penitrem F**	**618.4**	**600.0**	**10**	**16**	**140**
	618.4	582.2	20	30	140
	618.4	530.2	20	40	140
**Paxilline**	**436.2**	**418.2**	**10**	**16**	**220**
	436.2	182.1	20	45	220
	436.2	130.1	20	50	220
	436.2	167.1	20	92	220
**Cyclopiazonic acid**	337.1	196.1	10	31	80
	337.1	182.2	10	25	80
	337.1	167.1	10	61	80
